# Defective COX1 expression in aging mice liver

**DOI:** 10.1242/bio.059844

**Published:** 2023-03-02

**Authors:** Steffen Witte, Angela Boshnakovska, Metin Özdemir, Arpita Chowdhury, Peter Rehling, Abhishek Aich

**Affiliations:** ^1^ Department of Cellular Biochemistry, University Medical Center, Göttingen, 37073, Germany; ^2^ Cluster of Excellence “Multiscale Bioimaging: from Molecular Machines to Networks of Excitable Cells” (MBExC), University of Göttingen, Göttingen, 37075, Germany; ^3^ Fraunhofer Institute for Translational Medicine and Pharmacology ITMP, Translational Neuroinflammation and Automated Microscopy, Göttingen, 37075, Germany; ^4^ Max Planck Institute for Multidisciplinary Sciences, Göttingen, 37077, Germany

**Keywords:** Ageing, Mitochondria, Nanopore, Transcriptomics

## Abstract

Mitochondrial defects are associated with aging processes and age-related diseases, including cardiovascular diseases, neurodegenerative diseases and cancer. In addition, some recent studies suggest mild mitochondrial dysfunctions appear to be associated with longer lifespans. In this context, liver tissue is considered to be largely resilient to aging and mitochondrial dysfunction. Yet, in recent years studies report dysregulation of mitochondrial function and nutrient sensing pathways in ageing livers. Therefore, we analyzed the effects of the aging process on mitochondrial gene expression in liver using wildtype C57BL/6N mice. In our analyses, we observed alteration in mitochondrial energy metabolism with age. To assess if defects in mitochondrial gene expression are linked to this decline, we applied a Nanopore sequencing based approach for mitochondrial transcriptomics. Our analyses show that a decrease of the *Cox1* transcript correlates with reduced respiratory complex IV activity in older mice livers.

## Teaser

**Summary:** Mitochondrial function is severely affected in aging livers. Hence, the current study uses a Nanopore sequencing based approach for mitochondrial transcriptomics. The analyses show that a decrease of the Cox1 transcript correlates with reduced respiratory complex IV activity in older mice livers.

## INTRODUCTION

Mitochondria play a key role in cellular energy metabolism by providing the bulk of ATP to drive cellular activities. Moreover, they carry out additional important metabolic tasks such as the tricarboxylic acid cycle (TCA), β-oxidation of fatty acids, and ketogenesis. Due to their prokaryotic origin, mitochondria contain their own genome and a perfectly adapted transcriptomic machinery that differs substantially from those used to express the nuclear genome. The circular mitochondrial DNA (mtDNA) encodes 2 rRNAs, 22 tRNAs, and 11 mRNAs encoding 13 polypeptides that are core subunits of the oxidative phosphorylation (OXPHOS) complexes in the inner membrane (Anderson et al., 1981). Mitochondrial transcription, translation, and assembly of the OXPHOS complexes are highly complex processes. The mitochondrial gene expression process is coordinated by numerous nuclear-encoded, imported regulatory factors (D'Souza and Minczuk, 2018; Basu et al., 2020; Barshad et al., 2018). Since each mitochondrial-encoded polypeptide is part of an OXPHOS complex, mutations in mitochondrial DNA cause severe disease phenotypes (Taylor and Turnbull, 2005). In addition, accumulating mitochondrial DNA mutations have been linked to cellular aging processes (Münscher et al., 1993; Zhang et al., 1993; Nekhaeva et al., 2002; Taylor et al., 2003; McDonald et al., 2008). However, these dysfunction influence aging is highly controversial and not yet fully elucidated (Kauppila et al., 2018; Wolf, 2021; Vermulst et al., 2007).

Cellular senescence is considered as the process of general decline in cellular physiology leading to morbidity and mortality. Exploring further the connection between cellular aging and mitochondria, one finds that multifaceted pathways are linked with a mitochondrial contribution to aging (Sun et al., 2016). Conversely, age-related changes in the cell contribute to a severe decline in mitochondrial function as well (Chistiakov et al., 2014). The liver shows remarkable resilience to aging, but it is becoming increasingly clear that the liver mitochondria undergo similar cellular changes associated with aging as other tissues (Łysek-Gładysińska et al., 2021; Barazzoni et al., 2000). Aging causes changes to both the nuclear and mitochondrial genomes and the epigenome in liver (Hunt et al., 2019). Yet, also in hepatocytes, mitochondria fulfill crucial metabolic roles. In aged hepatocytes, mitochondria increase in size, show decreased membrane potential, and increased ROS production (Sugrue and Tatton, 2001; Sastre et al., 1996). Increased cell stress due to ROS production as a result of dysfunction of the OXPHOS system could also contribute to cellular aging. Because mitochondrial DNA, unlike nuclear DNA, lacks important repair and quality control mechanisms, mtDNA has been shown to be more susceptible to ROS (Miquel et al., 1980; Huang et al., 2020). However, it is not clear whether mtDNA mutations and mitochondrial malfunction are a cause, side effect, or consequence of aging. Therefore, more detailed insights into the role of mitochondrial DNA maintenance and transcription, and its adaptation to aging processes remain to be obtained.

Analyses of the nuclear transcriptome provided new insights into a variety of molecular pathways and cell type-specific aging markers (The Tabula Muris Consortium, 2020; Zhang et al., 2021). Gene expression studies in aging mouse liver revealed pathways of fibrosis and immune response to be upregulated while those of metabolism and cell cycle appear to be downregulated (Pibiri et al., 2015; White et al., 2015). Furthermore, multi-time-point transcriptomics in rats showed that the most prominent pathway downregulated with aging was oxidative phosphorylation and respiratory electron transport (Shavlakadze et al., 2019). Considering the importance of mitochondrial gene expression for the biogenesis of the OXPHOS system, it is important to examine the effect of ageing on this process. New technologies, such as the recent development of Nanopore sequencing, allow for fast genome- and transcriptome sequencing in a wide range of research areas (Wang et al., 2021). Especially, for future analyses of mitochondrial dysfunction, its adaptation to changing metabolic conditions, and involvement in human disease pathogenesis, a reliable and fast approach for monitoring of the mitochondrial transcriptome would represent a key technical asset. Therefore, we established a method for transcriptome analysis of mitochondrial mRNAs. Together with functional analyses of mitochondria, this approach provides new insights into mitochondrial changes during aging in healthy hepatocytes.

Considering a key role of mitochondria in aging and pathology, we investigated mitochondrial function in 12- and 65-week-old mice. For this, we established a Nanopore sequencing based method for mitochondrial transcriptome analyses. Our analyses revealed a robust decrease of *mt-COX1* transcript abundance resulting in reduced protein levels. Loss of the core subunit COX1 is in agreement with reduced complex IV activity in aged mice samples. Our findings provide new insights into how altered gene expression contributes to the functional decline of hepatic mitochondria.

## RESULTS

### Changes in mitochondrial membrane potential during ageing

**Fig. 1.**
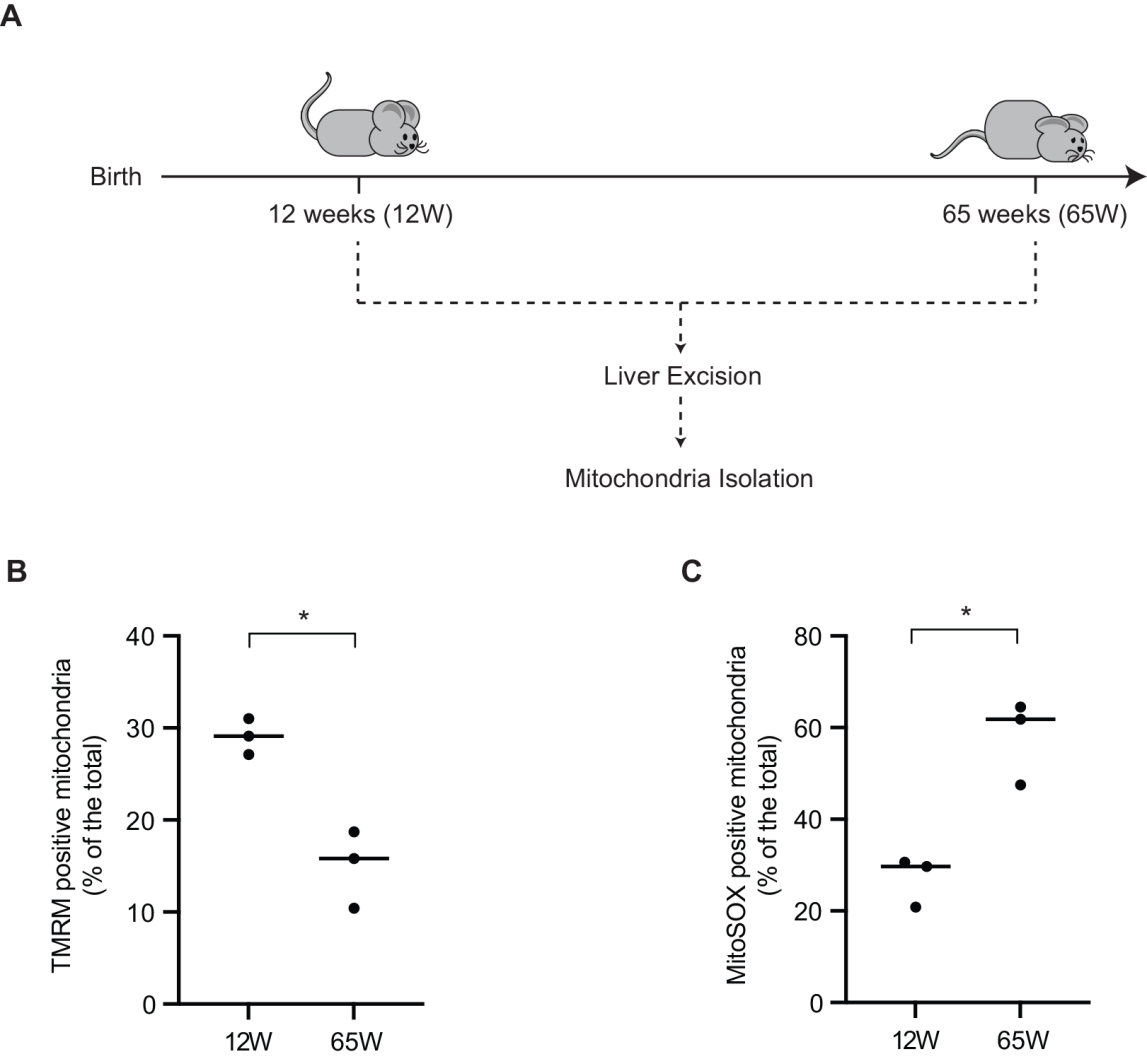
**Aged mitochondria show a decrease in membrane potential and increased oxidative stress**. (A) Overview of the experimental cohort of 12-week-old female mice compared with a cohort of 65-year-old mice and the procedures conducted. (B) Isolated mitochondria stained with TMRM to measure the mitochondrial membrane potential (column scatter plot showing mean and distribution of the biological replicates, * denotes *P*<0.05 from two-tailed unpaired *t*-test, *n*=3). (C) Isolated mitochondria stained with MitoSOX to measure the mitochondrial membrane potential (column scatter plot showing mean and distribution of the biological replicates, *P*<0.05 from two-tailed unpaired *t*-test, *n*=3).

Considering that hepatic cells are claimed to display to a certain extend resilience towards aging processes, we analyzed the correlation between different key parameters of mitochondrial physiology and age in the livers of young and aged mice 12 week (12 W) and 65 week (65 W), respectively (Fig. 1A). Livers excised from the 65 W mice showed a pale appearance. During the mitochondrial isolation the older livers also displayed a higher degree of fat layer abundance. The mitochondrial respiratory chain establishes a proton gradient across the inner membrane that drives ATP production by the *F*_1_*F*_o_ ATP synthase. To assess the mitochondrial membrane potential (Δσ), the isolated mitochondria from both experimental cohorts were stained with the membrane potential sensitive dye tetramethylrhodamin-methylester-perchlorat (TMRM). Applying the samples to flow cytometric analyses, we observed that the membrane potential was significantly reduced in the 65 W mice compared with that measured for the 12 W mice mitochondria (Fig. 1B). Similarly, when the isolated mitochondria were stained for superoxide production with fluorescent indicator MitoSOX, we found that superoxide levels were significantly increased in mitochondria from the 65 W mice compared to that of the 12 W mice (Fig. 1C). Based on these observations, we concluded that a decrease in the mitochondrial health parameters were apparent with increasing age.

### Mitochondrial energy metabolism changes with age

**Fig. 2.**
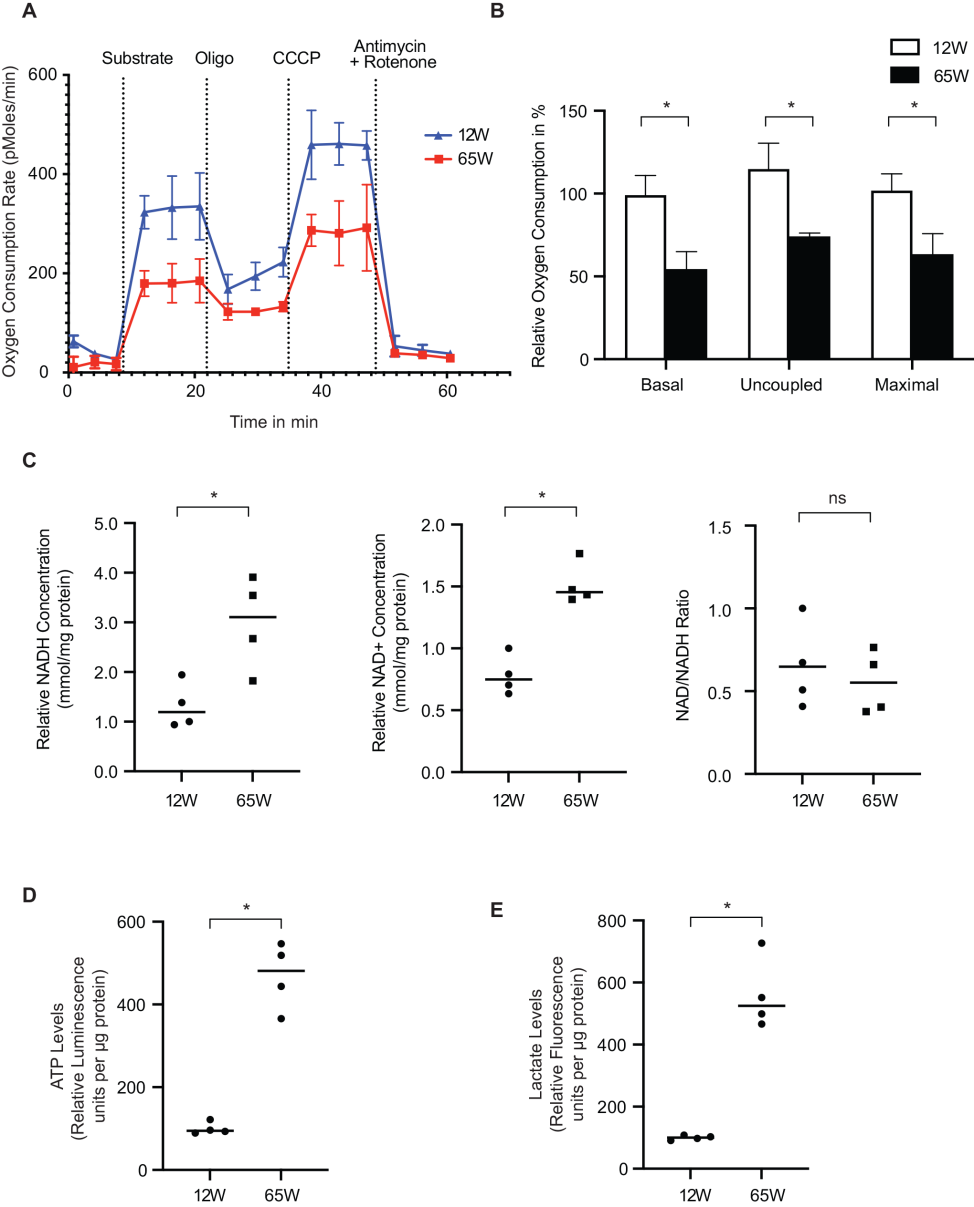
**Mitochondrial metabolism changes with age.** (A) Freshly isolated mitochondria from both cohorts were subjected to oxygen consumption rate measurement via a Seahorse Flux Analyzer to determine the respiratory capacity of the mitochondria (mean±s.e.m., *n*=4). (B) Basal respiration was calculated upon substrate injection; uncoupled respiration was calculated after oligomycin injection and maximal respiration was calculated after CCCP injection. Relative oxygen consumption per condition was calculated from the average of three measurements (mean±s.e.m., * denotes *P*<0.05 from two-tailed unpaired students *t*-test, *n*=4). (C) Total NAD and NADH were determined from equal amounts of tissue lysate form both cohorts using a NAD/NADH colorimetric assay kit (Sigma-Aldrich). NAD+ was calculated as the difference between NAD total and NADH. NAD/NADH ratio was also calculated. NAD+, NADH concentration (absorbance) and NAD/NADH ratio were normalized to the mean of the young mice samples (column scatter plot showing mean and distribution of the biological replicates, * denotes *P*<0.05 from two-tailed unpaired *t*-test, ns= non-significant, *n*=3). (D and E) Equal amounts of tissue lysate per cohort were used to determine ATP and Lactate levels respectively via fluorescence assay kits (Abcam) (column scatter plot showing mean and distribution of the biological replicates, * denotes *P*<0.05 from two tailed unpaired Students *t*-test, *n*=3).

A reduction in the mitochondrial membrane potential with increased superoxide production are indicative of dysfunction of the respiratory chain and concomitantly the oxidative phosphorylation process. Therefore, we assessed mitochondrial oxygen consumption by real time respirometry to determine if a decline in oxidative phosphorylation was apparent with age. In the presence of non-limiting amounts of ADP and substrate (state 3) actively respiring mitochondria reach a maximal physiological respiration. We found that state 3 respiration was lower in 65 W liver mitochondria as compared to those from 12 W old mice (Fig. 2A). The addition of oligomycin and CCCP to measure the uncoupled maximal respiration showed a similar difference. When we quantified averages obtained from three measurements in each case, we observed that the rate of oxygen consumption was significantly reduced under all conditions (Fig. 2B). Subsequently, we measured the NADH and NAD^+^ levels in liver tissue lysates. Interestingly, both metabolites were increased in the 65 W mitochondria (Fig. 2C). However, the NAD^+^/NADH ratio was similar in both age groups. Surprisingly, quantification of the ATP levels in the experimental cohort liver lysates showed a significantly higher levels of ATP in the 65 W mice samples (Fig. 2D). To assess if the increased amounts of ATP at steady state were the result of increased glycolysis, we also measured the lactate levels in the same samples and found them to be increased in the 65 W mice samples (Fig. 2E). Accordingly, our results indicated that in the liver samples from older mice the activity of the respiratory chain is decreased and cells display a highly glycolytic metabolism.

### Mitochondrial respiratory complex IV activity reduces with age

**Fig. 3.**
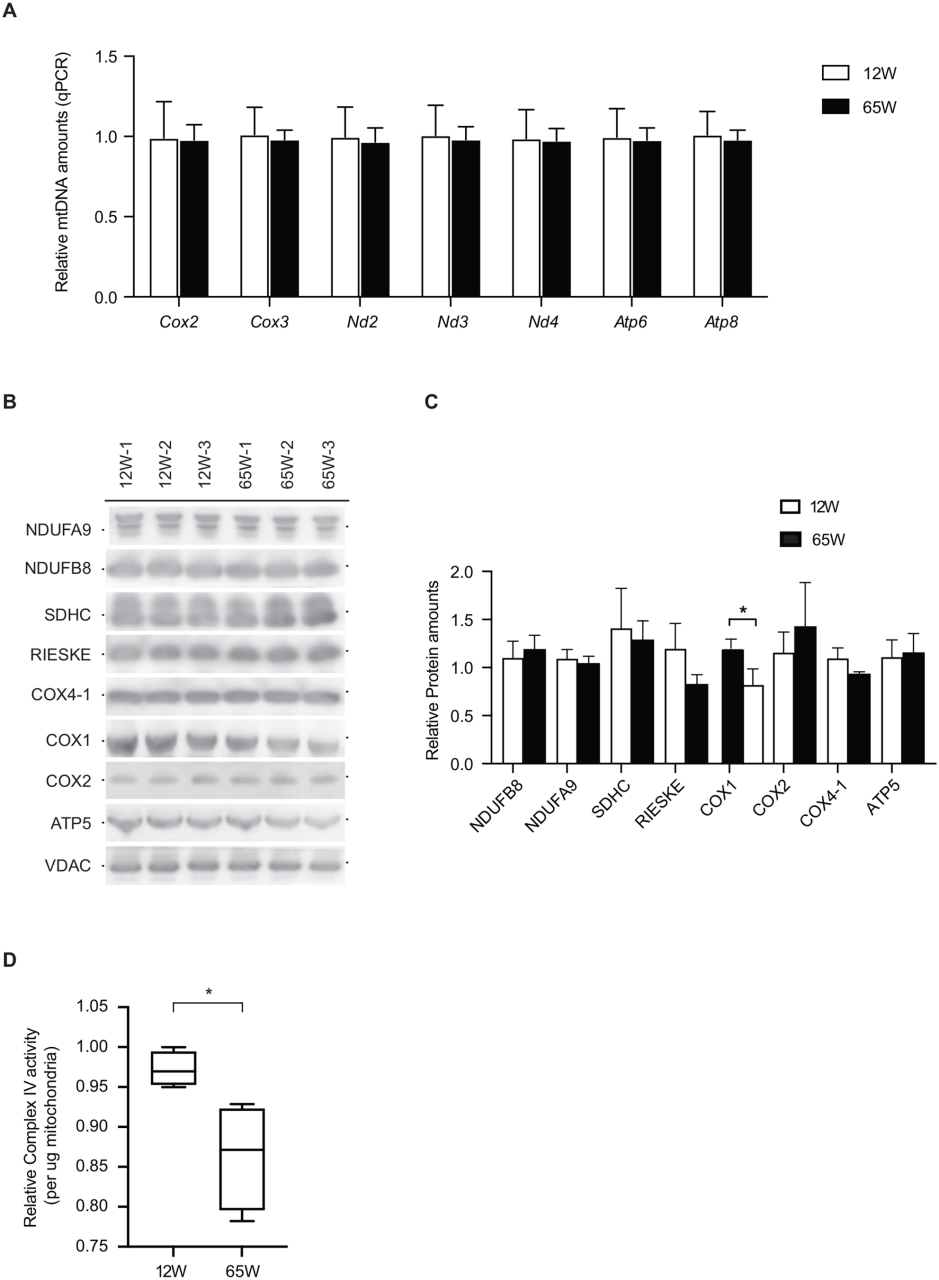
**OXPHOS complex IV decreases with age.** (A) Equal amounts of isolated mitochondria from both the cohorts subjected to mtDNA isolation and subsequent qPCR to test the different mt-genes in order to estimate the relative mtDNA amounts (mean±s.e.m., *n*=4). (B) Isolated mitochondria from both the cohorts subjected to Tris Tricine-SDS-Page and immunoblotting to check for OXPHOS (*n*=3). (C) Area under the curves were calculate with ImageJ and were normalized to Vdac (mean±s.e.m., * denotes *P*<0.05 from two-tailed unpaired Students *t*-test, *n*=3) (D) Complex IV activity was measured using activity assay microplate kit (Abcam). Complex IV activity was normalized to activity of a young mice samples and the mean±s.e.m. is shown (* denotes *P*<0.05 from two tailed unpaired *t*-test, *n*=3).

The reduced respiration observed in the mitochondria of 65 W mice led us to examine the amounts of mtDNA in the isolated mitochondria of the different mice. For this, we used equal amounts of purified mitochondria and treated the obtained DNA with RNAse to deplete mitochondrial RNA. Subsequently, we used real time PCR for a quantitative assessment of mtDNA. For complete coverage of mtDNA we used primers designed for several mitochondrial genes. These analyses showed that the mtDNA copy numbers were similar in both 12 W and 65 W mice (Fig. 3A). Next, we analyzed the protein levels of selected subunits of the mitochondrial OXPHOS complexes. Of the mitochondrial proteins addressed, only COX1 showed a reduction in the aged experimental cohort (Fig. 3B). Quantification of the blots confirmed a statistically significant reduction only in the levels of COX1 in the 65 W cohort (Fig. 3C). These findings suggested a selective effect on the cytochrome c oxidase (complex IV) of the respiratory chain. Accordingly, we assessed the activity of complex IV in the isolated mitochondrial. As expected from the steady state protein analyses, we found that the activity of complex IV was significantly reduced in the 65 week mitochondria samples, with a pronounced degree of variability compared to the 12 week samples (Fig. 3D). These findings on the activity of complex IV are in agreement with the reduced steady state levels of COX1.

### Mitochondrial transcriptome profiling by Nanopore sequencing

**Fig. 4.**
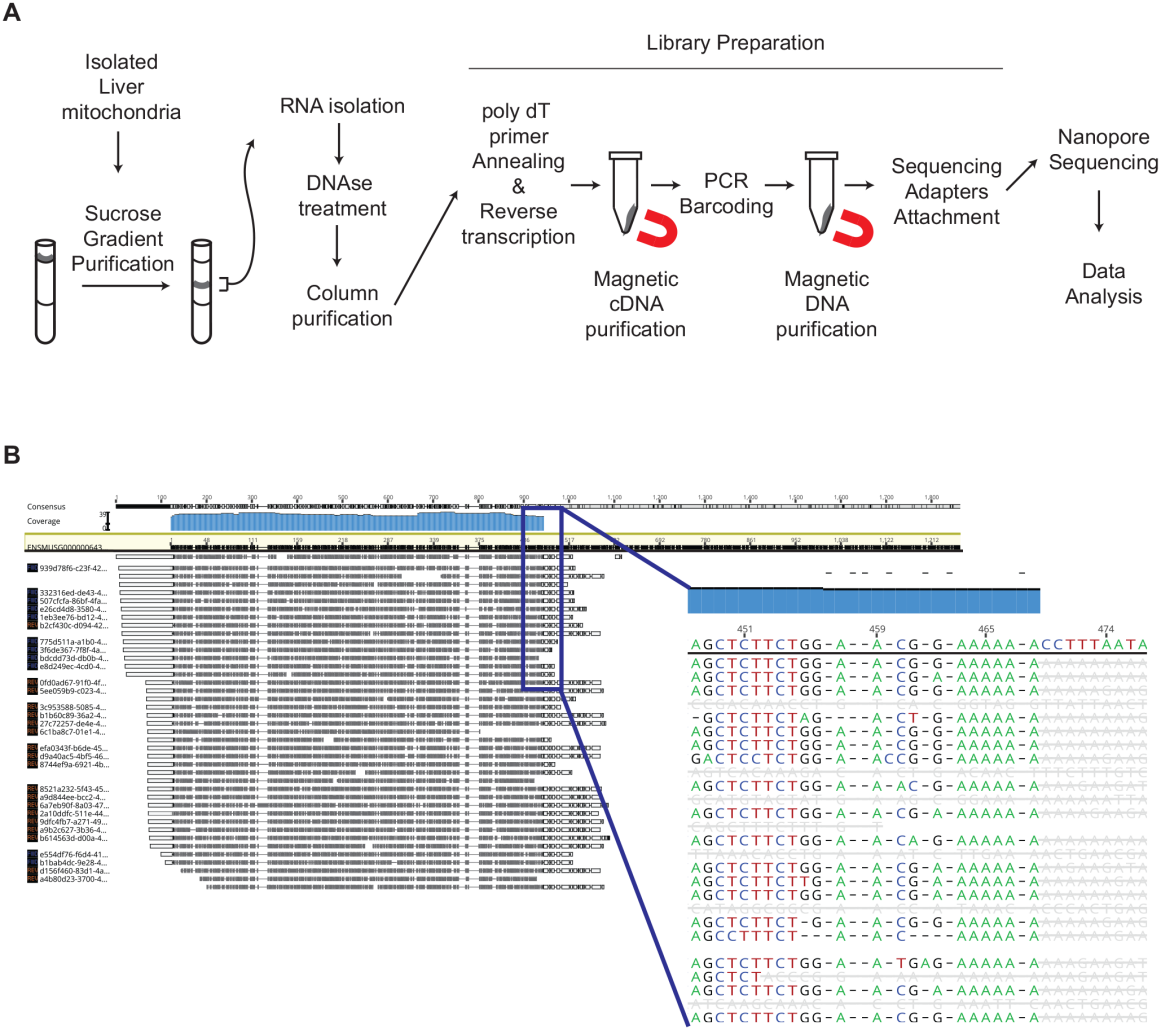
**OXPHOS complex IV decreases with age.** (A) Experimental overview of the purification of mitochondrial samples isolated from the livers of the experimental cohorts, RNA isolation, library preparation and Nanopore sequencing. (B) Representative image of mt-Rnr2 sequencing. Bam alignment file visualization with Geneious Prime shows the reference sequence highlighted in yellow and the consensus is marked in blue. Blue box shows a magnified view of the alignment in the region where the poly dT primer anneals during the library preparation.

To address as to why COX1 levels were reduced in liver mitochondria of 65 week old mice, we decided to assess mitochondrial RNA levels. Since the isolated crude mitochondrial fraction used for our analyses also contained microsomal membranes, we further enriched mitochondria by sucrose density centrifugations. Gradient-purified mitochondria were processed for RNA isolation and subsequent DNAse digestion was performed to avoid mtDNA contamination. The purified RNA was subjected to library preparation and Nanopore sequencing (Fig. 4A). The use of PCR barcoding allowed us to pool all samples and to analyze these together. Although we used a poly dT primer annealing approach for library generation, we were able to obtain sequencing reads for mitochondrial ribosomal RNAs, *Rnr1* and *Rnr2*. A detailed analysis of the reads showed that both RNAs contain a short internal stretch of poly A (Fig. 4B). Thus, the presence of an internal A-rich sequence enables poly dT primer annealing and subsequent recovery of *Rnr1* and *Rnr2*.

**Fig. 5.**
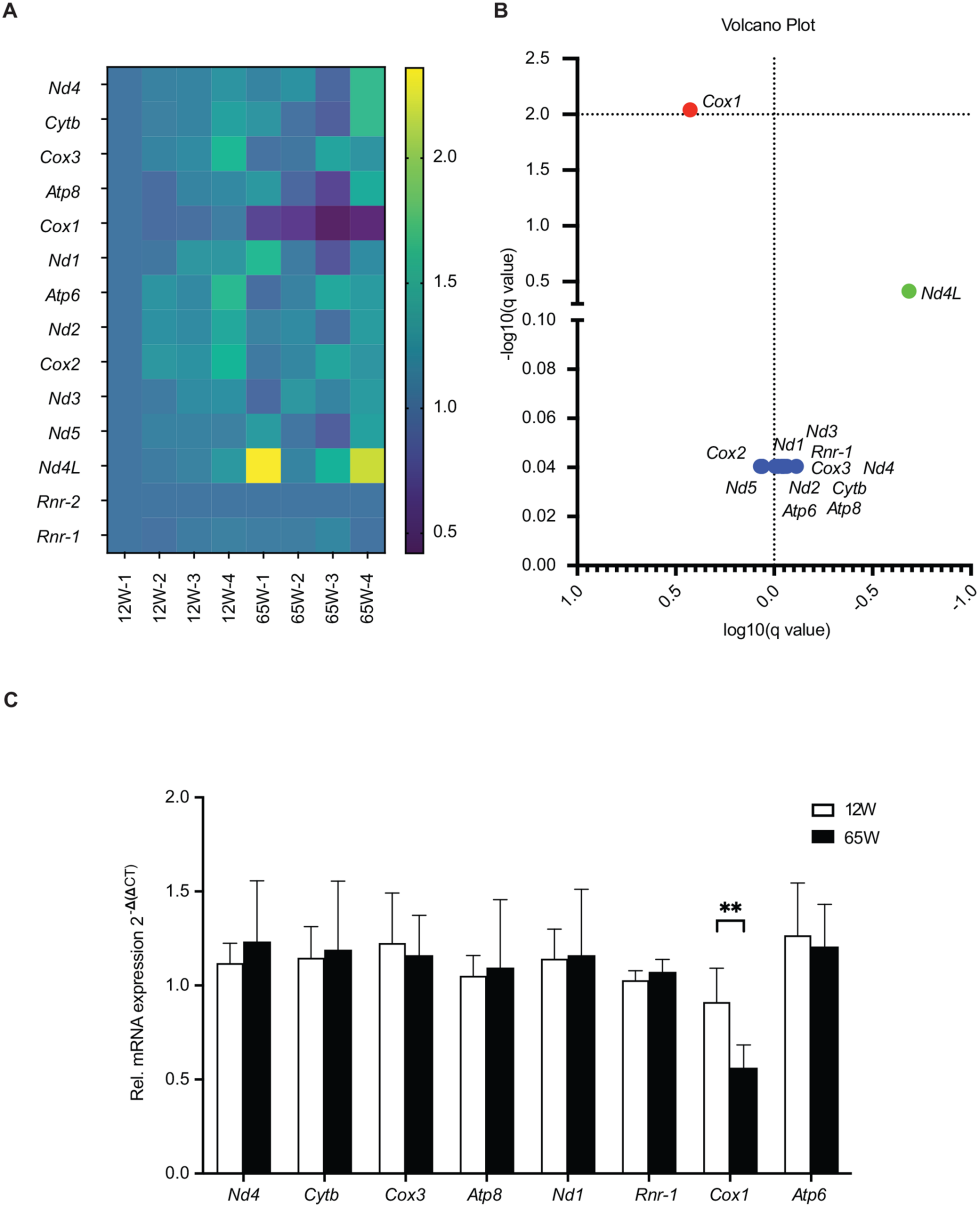
**OXPHOS complex IV decreases with age.** (A) Heatmap representation of the normalized transcript counts obtained from the differential gene expression workflow from Epi2Me Labs. Counts normalized to mt-Rnr2. Heatmap generated using GraphPad Prism 9 (*n*=4). (B) Volcano plot representation of (A) generated using GraphPad Prism 9 (*n*=4). Red denotes highly significant change. Green indicates a fold change over 0.5 and blue indicates no change. (C) Analysis of gene expression of mitochondrial transcripts in total mRNA isolated from 12 W and 65 W liver samples by qPCR. (means±s.e.m.s, ** denotes *P*<0.01 from two-tailed unpaired Students *t*-test, *n*=4).

Subsequently, the Nanopore sequencing results were analyzed using the Epi2me Labs differential gene expression pipeline (Oxford Nanopore Technologies). Normalization of the data to *Rnr2* was carried out and results displayed in a heatmap visualization (Table S2 for Mapped Trancript Counts). Interestingly, these analyses revealed that the *Cox1* transcript was strongly decreased in all the 65 W samples (Fig. 5A). Further statistical analysis showed that *Cox1* was the only transcript that was significantly different between mitochondria of the 65 W samples and those from the 12 W ones (Fig. 5B). Considering that we had incorporated barcodes using PCR in the experimental setup in order to multiplex the analysis, we decided to further exclude a PCR bias and inaccurate transcript number estimation. Therefore, we carried out qPCR analyses to confirm the reduction of the *Cox1* transcript by an alternative approach. Using this second strategy, we were able to confirm the reduction the *Cox1* transcript in liver mitochondria of 65 W samples (Fig. 5C). In conclusion, we observed a reduction of complex IV activity due to decrease in the levels of *Cox1* mRNA and protein. This finding agrees with the observed decline in OXPHOS activity and changes of mitochondrial health parameters that we observe in the liver mitochondria from mice samples with age.

## DISCUSSION

Cellular functions decline with age. In addition to DNA damage and increased ROS production, aging has been found to be associated with metabolic dysfunction (Houtkooper et al., 2011). During the aging process, energy demands, lipid metabolism and reaction conditions change. The adaptation of the OXPHOS system to these changing conditions appears to be a highly dynamic process that can be influenced by a variety of (external) factors such as diet or exercise (Perelló-Amorós et al., 2021; Chen et al., 2018; McCoin et al., 2019; Han et al., 2012). In this context, the liver represents a critical organ for energy and lipid metabolism and hepatic mitochondria are a central hub for oxidative phosphorylation, fatty acid metabolism, and ketogenesis. Therefore, to study the effects of adaptation of metabolic pathways to changing conditions hepatocytes represent a suitable cellular system. Moreover, the liver is the only visceral organ that can regenerate and is exposed to a high degree to changing metabolic conditions (Michalopoulos and Bhushan, 2021). Therefore, mitochondria need to adapt to metabolic challenges. They proliferate in a process of mitochondrial biogenesis through fusion and fission from pre-existing mitochondria (Ploumi et al., 2017). Thus, the transcription and translation of nuclear genes coding for mitochondrial proteins appears to be critical for many aspects of cellular physiology (Kotrys and Szczesny, 2020).

Here, we compared mitochondrial functions in the livers of young and old mice. Our analyses find that both ATP and lactate levels are significantly increased in liver in the aged group. This, in conjunction with the reduced oxygen consumption rates, indicates that the livers become increasingly glycolytic with age. Our finding is in line with previous studies which reported increased lactate levels and reduced glycolytic intermediates indicative of elevated anaerobic glycolysis (Houtkooper et al., 2011). Surprisingly, the NAD^+^ levels were found to be increased in this study. In contrast, other studies report a hepatic NAD^+^ deficiency in aged mice and humans (Zhou et al., 2016). However, in our analyses we found that the NAD^+^/NADH ratio remains unaffected in 65 W mice compared to the 12 W age cohort. In addition, we found a decrease in oxidative phosphorylation capacity of the mitochondria that are linked to a loss in COX1, the mitochondrial-encoded core subunit of the cytochrome c oxidase.

Third-generation sequencing has revolutionized many aspects of biology from genome assemblies, metagenomics, epigenetics and transcriptomics (Kraft and Kurth, 2019). Additionally, the use of the MinION sequencer (Oxford Nanopore Technologies) allows rapid, sensitive and real time long read sequencing of nucleic acids (Player et al., 2020; Tyler et al., 2018). Coupled with barcoding, it possible to further reduce the machine run time by pooling all the samples and replicates in a single run. Here we applied this experimental approach to address if a loss COX1 was linked to the availability of the corresponding transcript. For this, we purified mitochondria to eliminate cytosolic RNAs from the samples to be analyzed. This enrichment allowed for extensive sequencing of the mitochondrial transcripts alone. However, on the downside this approach reduced the number of housekeeping genes that could be used for normalizing the data during analysis. Therefore, we started with equal amounts of purified mitochondria for RNA extraction and subsequently used equal amounts of RNA for cDNA preparations and library generation. We chose to base our analyses on polyadenylated RNAs for the initiation for cDNA and library preparation. The presence of an internal poly A stretch, long enough for the oligo dT primer to bind, enabled the analysis of the mitochondrial *Rnr1* and *Rnr2*. Regarding the Cox1 transcript, the analyses revealed that in aged mitochondria the amount of the mRNA was specifically reduced in liver mitochondria of the 65 W mice while other transcripts were not affected. This reduction agrees with the observed decline in the respiratory activity.

## MATERIALS AND METHODS

Key resources are specified in table S1.

### Animals

Maintenance of all mice and their study were performed according to the guidelines from the German Animal Welfare Act and approved by the Landesamt für Verbraucherschutz und Lebensmittelsicherheit, Niedersachsen, Germany (AZ: 33.9-42502-04-14/1720). The animals were kept either in high barrier (SPF-specified pathogen free) areas in IVC (individually ventilated caging) on standard rodent chow to WT C57BL/6N mice, with restricted access for animal care staff only. The WT C57BL/6N mice were historically acquired from Charles River Laboratories, Research Models and Services, Germany GmbH, Sulzfeld. They were subsequently maintained at the animal facility at Max Planck Institute for Multidisciplinary Sciences, Göttingen till they reached appropriate age for experimentation.

### Mitochondrial isolation

The animals were sacrificed at the respective ages to isolate the liver. These were then homogenized using glass potters in 15 ml of Isolation Buffer (IB), containing 10 mM Tris-MOPS pH 7.4, 1 mM EGTA/Tris and 200 mM Sucrose. Nuclei, debris and unlyzed cells were removed by centrifugation at 700×***g***, 10 min, 4°C. Mitochondria were further pelleted at 7000×***g***, 10 min, 4°C. They were then washed and resuspended in Isolation Buffer and their protein concentration was determined using Bradford assay.

### Mitochondrial sucrose-gradient purification

To remove extramitochondrial nucleic acids from the isolated mitochondria, samples were first treated with 50 U Benzonase for 30 min, at 4°. Centrifugation (2×, 14,000×***g***, 10 min, 4°C) and resuspension in IB (composition as described in mitochondrial isolation, containing 2.5 mM EDTA) stopped the Benzonase activity. A sucrose gradient was prepared by placing 1 volume of isolation buffer containing 1.7 M sucrose into a centrifuge tube and overlaying this with 2 volumes of isolation buffer containing 1 M sucrose. Mitochondria were finally resuspended in 1 M sucrose isolation buffer and carefully loaded onto the gradient. After centrifugation for 25 min, 25,000 rpm (SW41Ti, Beckman Coulter) at 4°C, mitochondria were isolated from the interphase.

### Mitochondrial membrane potential measurement

Membrane potential measurement was conducted on isolated liver mitochondria (as described above) using a Flow Cytometer (FACSCanto, BD Biosciences). The mitochondria were resuspended in freshly prepared Analysis buffer (pH 7.0; 250 mM Sucrose, 20 mM Tris-MOPS, 100 µM Pi(K), 0.5 mM MgCl_2_, 5 mM Succinate, 2 µM Rotenone). 100 µM Tetramethylrhodamin-methylester-perchlorat, TMRM (Life Tech; T668) was added to all samples except the unstained control and the samples were incubated for 10 min at room temperature, protected from light. The measurement was done at Ex488/Em590 nm.

### Mitochondrial respiration analysis (Seahorse)

Oxygen Consumption Rate (OCR) of freshly isolated liver mitochondria (isolation as described above) was obtained via respirometry on a Seahorse XFe96 analyzer (Agilent; S7894-10000). The mitochondria were resuspended in Mitochondrial Assay (MAS) Buffer (pH 7.4; 70 mM Sucrose, 210 mM Mannitol, 5 mM HEPES, 1 mM EGTA, 10 mM KH_2_PO_4,_ 5 mM MgCl_2_, 0.5% BSA(w/v) to a concentration of 0.1 mg/ml. The mitochondria were aliquoted in the Seahorse XF96 Cell Culture Microplate (Agilent; 101085-004) and the plate was centrifuged at 2000×g for 5 min at room temperature. In port A of the Seahorse XFe96 Sensor Cartridge (Agilent; 101085-004) either a Pyruvate (0.1 M Pyruvate, 40 mM Succinate, 40 mM ADP in MAS buffer) or Succinate (100 µM Succinate, 40 µM ADP in MAS buffer) mix was provided as substrate for the mitochondria. 30 µM of Oligomycin, 40 µM of CCCP, 10 µM of Antimycin and Rotenone dissolved in MAS buffer were added in ports B, C, and D of the Sensor Cartridge respectively. A modified Mito Stress Test protocol was used for the measurement. The modifications were the removal of the Equilibration step for the Cell Plate to minimize the stress time on the isolated mitochondria and the addition of another Port injection to accommodate for the substrate.

### NAD/NADH measurement

Quantification of NAD^+^/NADH ratio in mouse liver tissue samples was done using a NAD^+^/NADH Quantification Kit (Sigma-Aldrich; MAK037). The protocol provided by the supplier was followed. NAD_total_ as well as NADH were detected measuring absorbance at 450 nm on a microplate reader (Synergy H1; BioTek; 8041000) following mechanical lysis and deproteination of the lysates. In order to measure NADH separately from the NAD_total_, NAD^+^ was thermally decomposed by a 30 min incubation at 60°C.

### ATP measurement

Measurement of total tissue ATP content was done using the ATP Assay Kit (Colorimetric/Fluorometric) (Abcam; ab83355) and as specified by the protocol from the supplier. In order to determine total ATP mouse liver tissue was lysed mechanically and deproteinized. Samples were incubated for 30 min at room temperature with the reaction mix in triplicates. The phosphorylation of glycerol resulting in a product detectable at 570 nm was measured using a microplate reader (Synergy H1; BioTek; 8041000).

### Lactate measurement

L-Lactate Assay Kit (Colorimetric/Fluorometric) (Abcam; ab65330) was used in order to detect lactate in mouse liver tissue lysate. The experiment was performed as instructed in the protocol provided by the supplier. Following mechanical lysis, the samples were deproteinized and assayed for 30 min. Lactate was detected calorimetrically at 570 nm. The measurement was done in triplicate and visualized using a microplate reader (Synergy H1; BioTek; 8041000).

### Mitochondrial DNA isolation

The QIAamp® DNA Blood Mini Kit was used to isolate mitochondrial DNA from mitochondria. The isolated mitochondria (see above) were diluted 1:50 in the provided sample buffer and the manufacturer's protocol was executed.

### RNA isolation and purification

The isolated and purified mitochondria were lysed in 1 ml TRIzol using the vendor's protocol. The samples were then treated with DNase I (ThermoFisher Scientific) following the manufacturer's instructions. For purification of the sample, the RNA Clean and Concentrator Kit (R1013, Zymo Research) was used. The RNA quality and concentration were measured using a Nanodrop 2000.

### cDNA synthesis and qPCR

cDNA was synthesized using the RevertAid First Strand cDNA Synthesis Kit (K1621; ThermoFisher Scientific), specifically using the Random Hexamer primers from the isolated RNA (described in the previous section) in a thermocycler (Labcycler Gradient, SensoQuest GmbH). The cDNA was used in quadruplicates for a Real Time PCR reaction with Sensi Mix SYBR Low-ROX Kit (QT625-05; Bioline) on a Quant Studio 6 Flex Real-Time PCR system (4485691; ThermoFisher Scientific). All the primer sequences of the primers used are available upon request.

### Tricine-SDS PAGE and western blot

Tricine-SDS PAGE was performed using standard methods. The samples were lysed in T-PER Tissue Protein Extraction Buffer (ThermoFisher Scientific; 78510). The gels used were gradient 10-18% gels. Western blotting was preformed using standard semi-dry transfer.

### Cytochrome c oxidase activity assay

The Complex IV Rodent Enzyme Activity Microplate Assay Kit (Abcam; ab109911) was used to assess the activity of the Cytochrome c Oxidase in isolated mouse liver mitochondria. The procedure was performed as instructed by the provider. The principle of the assay is the use of immunoprecipitated mitochondrial complex IV, whose activity gets determined by the oxidation of Cytochrome c. A microplate reader (Synergy H1; BioTek; 8041000) was used to measure the absorbance of cytochrome c, which turns colorless due to its oxidation and is detectable at 550 nm.

### Library preparation

Total mitochondrial RNA was purified and concentrated on an RNA Clean Concentrator™-5 column (Zymo Research, Irvine, CA, USA). cDNA libraries were prepared from a mix of 50 ng RNA according to the Oxford Nanopore Technologies (Oxford Nanopore Technologies Ltd, Oxford, UK) protocol ‘DNA-PCR Sequencing’ with a 14 cycles PCR (8 min for elongation time). ONT adapters were ligated to 650 ng of cDNA.

### Nanopore sequencing and analysis

Nanopore libraries were sequenced using a MinION Mk1b with R9.4.1 flowcells. The data were generated and basedcalled using MinKNOW (Version 21.11.9). The fastq files were then uploaded to and analyzed using the Epi2Me Labs (Version 1.1) Differential Gene Expression workflow. Transcript counts obtained were processed for heatmap and volcano plot generation using Prism 9 software (GraphPad Software, San Diego, CA, USA).

### Statistical analysis

Significant differences between means of two (or more) sets of data were analyzed using either unpaired *t*-tests or ANOVA tests in Prism 9 software (GraphPad Software, San Diego, CA, USA, unless otherwise noted.

### Ethics approval and consent to participate

Maintenance of all mice and their study were performed according to the guidelines from the German Animal Welfare Act and approved by the Lower Saxony government office ethic committee, Landesamt für Verbraucherschutz und Lebensmittelsicherheit, Niedersachsen, Germany (AZ: 33.9-42502-04-14/1720). All methods are reported in accordance with ARRIVE guidelines for the reporting of animal experiments.

## Supplementary Material

10.1242/biolopen.059844_sup1 Supplementary information
