# Supplementary material for: Defective COX1 expression in aging mice liver
Source: Biol Open. 2023 Mar 2;12(3):bio059844. doi: 10.1242/bio.059844 (PMC10003073; doi:10.1242/bio.059844)
Supplement: Supplementary information [file biolopen-12-059844-s1.pdf]

Table S1. Key Resource Table

Click here to download Table S1

Table S2. Mapped Transcript Counts

Click here to download Table S2

Biology Open • Supplementary information
